# Oxidative Stress in Pediatric Obstructive Sleep Apnea Syndrome: A Narrative Review

**DOI:** 10.3390/antiox15101313

**Published:** 2026-10-12

**Authors:** Vasile Valeriu Lupu, Emil Anton, Ileana Ioniuc, Oana Raluca Temneanu, Irina Tarnita, Leonard Iosif Pertea, Elena Cristina Mitrofan, Magdalena Cuciureanu, Alice Grudnicki, Manuel Florin Rosu, Sorana Caterina Anton, Costica Mitrofan, Silvia Fotea, Alin Horatiu Nedelcu, Ancuta Lupu

**Affiliations:** 1 Grigore T. Popa University of Medicine and Pharmacy, 700115 Iasi, Romania; vasile.lupu@umfiasi.ro (V.V.L.); emil.anton@umfiasi.ro (E.A.); ileana.ioniuc@umfiasi.ro (I.I.); leonard.pertea@umfiasi.ro (L.I.P.); mag.cuciureanu@umfiasi.ro (M.C.); alice.azoicai@umfiasi.ro (A.G.); florin.rosu@umfiasi.ro (M.F.R.); sorana.anton@umfiasi.ro (S.C.A.); costica.mitrofan@umfiasi.ro (C.M.); alin.nedelcu@umfiasi.ro (A.H.N.); ancuta.ignat1@umfiasi.ro (A.L.); 2 Clinic of Pulmonary Disease, 700115 Iasi, Romania; criselend@yahoo.com; 3 Clinical Medical Department, Faculty of Medicine and Pharmacy, “Dunarea de Jos” University of Galati, 800008 Galati, Romania; silvia.fotea@ugal.ro

**Keywords:** pediatric obstructive sleep apnea, oxidative stress, intermittent hypoxia, reactive oxygen species, oxidative biomarkers, inflammation, endothelial dysfunction, hypoxic burden, children

## Abstract

Pediatric obstructive sleep apnea syndrome (OSAS) is a common sleep-related breathing disorder increasingly recognized as a multisystem disease rather than an isolated disorder of the upper airway. Recurrent episodes of upper airway obstruction generate intermittent hypoxia, reoxygenation, and sleep fragmentation, promoting excessive production of reactive oxygen and nitrogen species and disruption of antioxidant defenses. This narrative review summarizes current evidence regarding the mechanisms, biomarkers, and clinical consequences of oxidative stress in pediatric OSAS. Intermittent hypoxia activates several redox-sensitive pathways, including NADPH oxidases, hypoxia-inducible factor-1α, nuclear factor-κB, mitochondrial dysfunction, and endoplasmic reticulum stress, thereby creating a bidirectional relationship between oxidative stress and systemic inflammation. Pediatric studies have demonstrated alterations in lipid peroxidation products, glutathione redox balance, urinary 8-hydroxy-2′-deoxyguanosine, isoprostanes, NOX2-related markers, and inflammatory mediators. These abnormalities are associated with endothelial dysfunction and cardiovascular, neurocognitive, metabolic, hepatic, renal, and immune consequences, and several may improve following effective treatment. Importantly, the magnitude of oxidative and end-organ injury does not consistently parallel the apnea-hypopnea index, suggesting that hypoxic burden, obesity, genetic susceptibility, antioxidant capacity, and disease duration influence individual biological vulnerability. Although several oxidative biomarkers show potential for disease characterization and treatment monitoring, methodological heterogeneity and the absence of standardized pediatric reference values currently limit their clinical application. Future integration of polysomnographic hypoxic-burden metrics with oxidative, inflammatory, metabolomic, and genomic biomarkers may improve risk stratification and facilitate more personalized management of pediatric OSAS.

## 1. Introduction

Obstructive sleep apnea syndrome (OSAS) is one of the most common sleep-related breathing disorders in childhood. It is characterized by recurrent partial or complete upper airway obstruction during sleep despite persistent respiratory effort, resulting in intermittent hypoxemia, hypercapnia, repeated arousals, and disrupted sleep architecture [1,2,3]. Unlike in adults, where excessive daytime sleepiness dominates the clinical picture, pediatric OSAS more often presents with disrupted growth, behavioral change, and neurocognitive impairment, reflecting the vulnerability of the developing brain and endocrine system to fragmented sleep and intermittent hypoxia. Risk factors also differ from those in adults: adenotonsillar hypertrophy is the leading anatomical contributor in otherwise healthy children, whereas obesity, craniofacial abnormalities, neuromuscular disease, prematurity, and genetic syndromes substantially increase susceptibility and severity [2,3,4]. Population-based estimates vary depending on the diagnostic criteria and recording method used, which contributes to the range of reported prevalence figures across different countries and cohorts [1,2]. Prevalence is estimated at 1–5% [1,2], rising sharply among children with obesity, Down syndrome, or craniofacial malformations, and the disorder remains widely underdiagnosed, as nocturnal symptoms are often overlooked while daytime behavioral changes are misattributed to primary behavioral disorders [1,2,3,4,5]. This diagnostic delay is clinically significant, since the interval between symptom onset and treatment may correspond to a period of ongoing, cumulative oxidative and inflammatory exposure during a developmentally sensitive window.

Untreated pediatric OSAS is associated with neurocognitive and behavioral impairment, growth disturbance, metabolic dysregulation, systemic inflammation, endothelial dysfunction, and altered blood-pressure regulation; persistence into adolescence is linked to long-term cardiovascular risk [4,6,7,8,9,10]. Importantly, the severity of these complications correlates poorly with the apnea-hypopnea index, suggesting that individual inflammatory, metabolic, and redox responses—rather than respiratory-event count alone—determine end-organ vulnerability [4,6,7,8,9,10]. This has direct clinical implications: a child classified as having “mild” disease by conventional polysomnographic criteria may still carry substantial biological risk if hypoxic episodes are prolonged, frequent, or occur alongside obesity or a genetic predisposition to impaired antioxidant capacity, whereas a child with a numerically higher apnea-hypopnea index may show comparatively little measurable end-organ injury.

Among the mechanisms proposed to explain this variability, oxidative stress has emerged as a central pathophysiological link between recurrent airway obstruction and multisystem dysfunction. Repeated cycles of desaturation and reoxygenation in OSAS resemble ischemia–reperfusion injury, driving excessive reactive oxygen species (ROS) production through mitochondrial and enzymatic sources such as NADPH oxidase and xanthine oxidase, and activating redox-sensitive pathways including HIF-1α and NF-κB [11,12]. Direct pediatric evidence supports this link: increased lipid peroxidation correlates with hypoxia severity and cardiovascular morbidity [13], NADPH oxidase activity is increased alongside reduced nitric oxide bioavailability [14], and endothelial function is reduced but may improve after adenotonsillectomy, supporting a potentially reversible relationship between upper airway obstruction and early vascular injury [15]. Nonetheless, residual endothelial, inflammatory, or oxidative abnormalities may persist in some patients, particularly those with obesity, severe disease, or persistent OSAS after treatment [5,16]. ROS may further damage lipids, proteins, and nucleic acids, and experimental intermittent-hypoxia models show oxidative injury in brain regions governing learning and behavior, offering a mechanistic explanation for neurocognitive sequelae [17]. Oxidative stress and inflammation interact bidirectionally, creating self-amplifying cycles that may explain persistent organ dysfunction even after respiratory parameters improve [11,12,17].

Although numerous oxidative biomarkers have been studied, pediatric findings remain heterogeneous because of differences in age, obesity status, disease severity, and analytical technique. It therefore remains uncertain whether these molecules merely reflect hypoxic exposure or can also predict severity and treatment response. This heterogeneity is compounded by the fact that most mechanistic and biomarker studies were originally conducted in adults, so pediatric extrapolation must account for children’s distinct growth-related metabolic demands, differing fat distribution, and generally shorter disease duration—all of which may modulate the magnitude and detectability of redox imbalance. This narrative review synthesizes current evidence on oxidative stress in pediatric OSAS, covering the molecular pathways activated by intermittent hypoxia, the principal biomarkers investigated in children, and the contribution of redox imbalance to cardiovascular, metabolic, and neurocognitive morbidity, before concluding with knowledge gaps and directions for biomarker-guided management. Given the breadth of organ systems potentially affected, the review first establishes the clinical and diagnostic context of pediatric OSA, then covers the fundamental biochemistry of reactive oxygen and nitrogen species, the specific mechanisms through which intermittent hypoxia generates oxidative injury, the biomarkers used to detect this injury in children, and finally the downstream organ-specific consequences that make this pathway clinically relevant.

### Literature Search Strategy

A narrative literature search was conducted using PubMed/MEDLINE to identify studies addressing oxidative stress in pediatric obstructive sleep apnea. Search terms included combinations of “pediatric obstructive sleep apnea”, “children”, “sleep-disordered breathing”, “oxidative stress”, “reactive oxygen species”, “intermittent hypoxia”, “NADPH oxidase”, “oxidative biomarkers”, “lipid peroxidation”, “8-isoprostane”, “8-hydroxy-2′-deoxyguanosine”, “inflammation”, “endothelial dysfunction”, “neurocognitive dysfunction”, and “organ damage”. The search covered publications and additional relevant articles that were identified through screening of reference lists. Priority was given to original pediatric studies, systematic reviews, meta-analyses, and recent mechanistic studies directly relevant to pediatric OSA. Adult, animal, and in vitro studies were included when pediatric evidence was limited and when they provided relevant mechanistic context. Because this was designed as a narrative review, study selection was not performed according to a formal systematic-review protocol, and no quantitative meta-analysis was undertaken.

## 2. Clinical Background of Pediatric Obstructive Sleep Apnea

**Figure 1:**
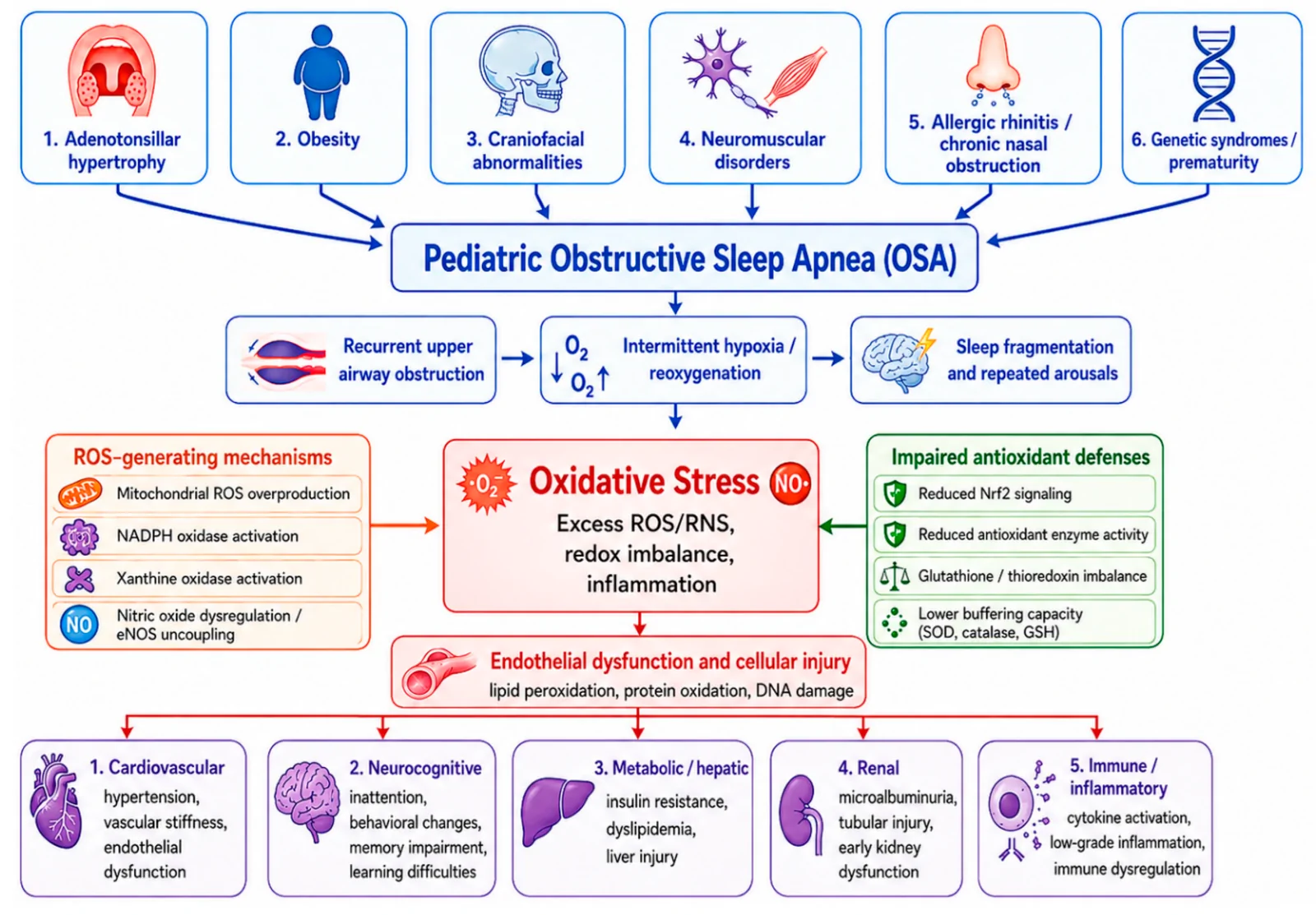
Causes of pediatric obstructive sleep apnea and the systemic effects of oxidative stress. Abbreviations: OSA, obstructive sleep apnea; ROS, reactive oxygen species; RNS, reactive nitrogen species; NADPH, nicotinamide adenine dinucleotide phosphate; eNOS, endothelial nitric oxide synthase; NO, nitric oxide; Nrf2, nuclear factor erythroid 2-related factor 2; SOD, superoxide dismutase; GSH, reduced glutathione; DNA, deoxyribonucleic acid; ↑O2-increasing oxygen (reoxigenation); ↓O2-deacreasing oxigen. The figure was generated with the assistance of ChatGPT 5.6 Sol (OpenAI, Ohio, DE, USA) and subsequently reviewed and edited by the authors for scientific accuracy.

Pediatric OSA arises from anatomical upper airway narrowing combined with impaired neuromuscular compensation during sleep, when the physiological reduction in dilator-muscle activity increases pharyngeal collapsibility [18,19,20]. Adenotonsillar hypertrophy is the principal cause in otherwise healthy children, although tonsillar size correlates poorly with severity: some children with markedly enlarged tonsils show only primary snoring, whereas others with modest lymphoid enlargement develop severe disease. Airway collapsibility, craniofacial morphology, and local inflammatory processes also contribute [19,21,22]. Adenotonsillectomy remains first-line treatment, but persistent disease is common, particularly in children with obesity, craniofacial abnormality, or genetic syndromes, confirming that this is frequently a multifactorial disorder [20,23]. Obesity has become an increasingly important, often coexisting risk factor, acting mechanically through fat deposition around the neck, tongue, and lateral pharyngeal walls, which narrows the airway and reduces lung volume, and metabolically through chronic inflammation, insulin resistance, and altered adipokine signaling; this creates a bidirectional relationship with OSA and oxidative stress, and obesity is among the strongest predictors of residual disease after surgery [24,25,26]. Craniofacial abnormalities—including Pierre Robin sequence, craniosynostosis, cleft palate, and achondroplasia—may cause multilevel obstruction involving both the nasopharynx and hypopharynx that surgery alone cannot correct, occasionally requiring cephalometric or endoscopic assessment to localize the site of collapse [27,28]. Neuromuscular disorders such as Duchenne muscular dystrophy and spinal muscular atrophy produce pharyngeal and respiratory-pump weakness with REM-predominant hypoventilation that may precede daytime symptoms by years, warranting continuous carbon dioxide monitoring during polysomnography, since standard apnea-hypopnea metrics may underestimate the true burden of hypoventilation [29,30]. Genetic syndromes, particularly Down syndrome and the mucopolysaccharidoses, combine anatomical narrowing from macroglossia and midface hypoplasia with hypotonia and lymphoid hypertrophy; because parental symptom report is notably unreliable in this population, many pediatric guidelines recommend routine polysomnographic screening irrespective of reported symptoms [31,32,33] (Figure 1).

Clinical presentation is heterogeneous and, unlike in adults, is dominated less by daytime sleepiness than by habitual snoring, labored breathing, witnessed pauses in breathing, snorting or gasping, and behavioral, emotional, and neurocognitive disturbance [34,35,36]. Some children adopt compensatory sleep postures, such as neck hyperextension, to maintain airway patency; unusual sleep postures, enuresis, and other parasomnias may also occur [37]. Hyperactivity and attention deficits resembling ADHD are common and do not correlate linearly with the apnea-hypopnea index, suggesting that intermittent hypoxia and sleep fragmentation affect neurodevelopment through pathways only partly captured by conventional respiratory severity metrics [38,39]. Academic performance may improve after treatment, providing indirect evidence that at least part of the neurobehavioral burden of OSA is modifiable rather than fixed [40]. Growth impairment, historically attributed to increased energy expenditure from labored breathing and reduced growth-hormone secretion during fragmented sleep, was characteristic of earlier pediatric OSA cohorts; in most contemporary series, however, obesity now predominates among affected children, reflecting a broader epidemiological shift in the pediatric OSA population over the past two decades [41]. Subclinical cardiovascular changes—elevated blood pressure, loss of nocturnal dipping, endothelial dysfunction—may already be present [42], and metabolic derangement is amplified when OSA coexists with obesity [25,26]. Because high-risk groups such as children with neuromuscular disease or Down syndrome may show minimal parental symptom recognition, the absence of classic symptoms does not exclude OSA [29,31]. History and examination alone cannot reliably confirm diagnosis or severity, which is why objective testing remains indispensable even when clinical suspicion appears low [43].

Attended polysomnography remains the diagnostic reference standard, quantifying the obstructive apnea-hypopnea index alongside desaturation, arousals, and ventilatory parameters used for severity grading; pediatric thresholds are considerably lower than adult ones, since even a modest number of respiratory events per hour may carry clinical significance in children, and testing is indicated especially in complex or persistent cases [44,45]. This lower threshold reflects the developing brain’s heightened sensitivity to intermittent hypoxia and sleep fragmentation compared with the mature adult brain, and underscores why pediatric and adult OSA, despite sharing a common respiratory mechanism, are managed as clinically distinct entities. Home sleep testing offers a more accessible alternative, allowing assessment in the child’s usual sleep environment, but it typically lacks EEG and therefore calculates respiratory indices from total recording time rather than true sleep time, risking underestimation of severity; it is also unsuitable for infants or children with complex comorbidity [46]. Screening questionnaires—the Pediatric Sleep Questionnaire and OSA-18—assist risk stratification and quality-of-life assessment but correlate poorly with polysomnographic severity [47,48,49]. Combining these tools may improve triage but cannot replace a complete sleep study. Critically, the apnea-hypopnea index alone provides limited insight into cumulative hypoxic burden or individual susceptibility to oxidative and inflammatory injury—the gap the following sections address. This limitation is particularly relevant for clinical decision-making, since treatment thresholds and prioritization for surgery are still largely based on the apnea-hypopnea index, despite mounting evidence that hypoxic burden, sleep fragmentation, and individual redox capacity may better predict which children face the greatest risk of long-term morbidity [50].

Differential diagnosis in children with suspected OSA also requires distinguishing obstructive events from central apneas, periodic limb movements, and other primary sleep disorders that can produce overlapping daytime symptoms [44,45]. Comorbid conditions such as allergic rhinitis, asthma, and gastroesophageal reflux may exacerbate upper airway inflammation and worsen obstructive symptoms, further complicating clinical assessment [34,36]. Because polysomnography capacity remains limited in many pediatric centers, triage strategies that combine clinical history, screening questionnaires, and, where available, oximetry or limited-channel studies are increasingly used to prioritize children for full studies, although none of these approaches can substitute for attended polysomnography when the diagnosis remains uncertain or when surgical planning is required [46,47,48,49]. This diagnostic complexity reinforces the rationale for biomarker-based approaches capable of capturing biological injury independently of the specific respiratory-event pattern recorded on a single night of testing [50].

## 3. Oxidative Stress: Biological Background

Oxidative stress reflects an imbalance between reactive-species production and antioxidant buffering capacity; at low, tightly regulated concentrations (“oxidative eustress”), these molecules serve as physiological signaling agents, whereas excess or prolonged exposure (“oxidative distress”) damages lipids, proteins, and nucleic acids [51]. Reactive oxygen species (ROS) include the superoxide anion, hydroxyl radical, hydrogen peroxide, singlet oxygen, and lipid-derived radicals, generated through sequential one-electron reduction of oxygen and, via the Fenton reaction with transition metals, forming the highly reactive hydroxyl radical, which reacts almost instantaneously with virtually any nearby biomolecule [51,52]. Mitochondria are a major ROS source through electron leakage from respiratory-chain complexes I and III, an effect substantially amplified during hypoxia-reoxygenation: repeated cycles progressively impair mitochondrial proteins, lipids, and mitochondrial DNA, thereby generating additional ROS in a self-perpetuating loop [53]. NADPH oxidases (the NOX1–5 and DUOX1/2 family) constitute a second major, tissue-regulated source, with NOX2 prominent in phagocytic and endothelial cells and NOX4 generating mainly hydrogen peroxide, with both physiological and pathological roles depending on its cellular context [54]. Unlike mitochondrial ROS, which arise largely as an unintended by-product of electron transport, NADPH oxidase activity is a deliberately regulated enzymatic process, making it a particularly attractive therapeutic target for modulating oxidative injury without broadly suppressing normal mitochondrial respiration [54]. Reactive nitrogen species arise mainly from nitric oxide synthase isoforms, of which the neuronal, endothelial, and inducible forms differ substantially in their regulation, tissue distribution, and typical output [55,56,57]. Superoxide reacting with nitric oxide forms peroxynitrite, which oxidizes thiols, initiates lipid peroxidation, damages mitochondrial enzymes, and nitrates proteins—effects amplified when endothelial nitric oxide synthase becomes “uncoupled” under oxidative conditions [55,56,57]. Lipid peroxidation represents one of the major consequences of excessive ROS generation. The process begins when a reactive radical abstracts a hydrogen atom from a polyunsaturated fatty acid, generating a lipid radical that rapidly reacts with molecular oxygen to form a lipid peroxyl radical. This radical can then attack neighboring fatty acids, producing lipid hydroperoxides and propagating the oxidative chain reaction [58]. Arachidonic acid, particularly when incorporated into membrane phospholipids, is highly susceptible to this process. Its non-enzymatic, free-radical-mediated oxidation generates F2-isoprostanes, including 8-isoprostane, independently of cyclooxygenase activity [59]. In parallel, decomposition of lipid hydroperoxides derived from arachidonic acid and other polyunsaturated fatty acids produces reactive aldehydes such as malondialdehyde (MDA) [58]. Thus, MDA reflects broader polyunsaturated-fatty-acid peroxidation, whereas F2-isoprostanes are more specific indicators of free-radical-mediated arachidonic-acid oxidation [58,59]. Endogenous antioxidant defenses counterbalance these processes: superoxide dismutase isoforms, including the extracellular SOD3 important in the vascular wall [60]; the glutathione system, in which reduced glutathione is oxidized to GSSG and regenerated via glutathione reductase using NADPH [61]; and the thioredoxin system, which together with thioredoxin reductase and NADPH maintains protein thiols in a reduced state [62]. Together, these enzymatic and non-enzymatic systems normally operate with substantial reserve capacity, so measurable oxidative damage typically reflects a sustained or repeated challenge—such as the recurrent hypoxia-reoxygenation cycles of OSA—rather than a single transient insult [53,63]. Exogenous antioxidant availability may also modulate the systemic response to oxidative challenge; in untrained subjects, pre-administration of vitamin C attenuated exercise-induced changes in oxidative-stress markers, including malondialdehyde and glutathione peroxidase activity [52].

The maturation of endogenous antioxidant systems is not static across childhood: total antioxidant capacity and the relative contribution of individual enzymatic defenses change with age, pubertal status, and body composition, which may partly explain why oxidative-biomarker levels reported in pediatric cohorts do not always mirror those described in adults with OSA [51,61,62]. Because children are also undergoing rapid somatic growth, their baseline metabolic and redox demands differ from those of adults, which could alter both the production and buffering of reactive species during intermittent hypoxia. This developmental variability underscores the importance of using age-matched controls and accounting for pubertal stage when interpreting oxidative-stress biomarkers in pediatric OSA research [60,61].

Children with OSA show increased circulating soluble NOX2-derived peptide (sNOX2-dp) and serum 8-iso-prostaglandin F2α, together with impaired flow-mediated dilation (FMD). Following adenotonsillectomy, both oxidative-stress markers decrease significantly, while FMD improves, supporting at least partial reversibility of these abnormalities [55]. In children with OSA, 8-isoprostane measured in exhaled breath condensate is increased compared with primary snorers and healthy controls and correlates with polysomnographic severity, whereas FeNO does not show a comparable association [57]. Urinary 8-hydroxy-2′-deoxyguanosine, a marker of oxidative DNA damage, correlates with cognitive dysfunction [63]. Early pediatric studies by Tauman et al. demonstrated increased lipid peroxidation linked to intermittent hypoxia and cardiovascular morbidity [64], while Tiboc et al. subsequently identified increased NOX2 activation and serum isoprostanes in children with sleep-disordered breathing, with improvement after adenotonsillectomy, showing an interaction between oxidative stress, inflammation, disease severity, and treatment-related response [65]. Taken together, these studies provide preliminary evidence that oxidative stress associated with pediatric OSA can be detected in blood, urine, and exhaled breath using relatively accessible and minimally invasive approaches [55,57,63,64,65]. Some biomarkers have been associated with polysomnographic disease severity, and changes following treatment have been reported in small pediatric cohorts [55,57,65]. However, circulating or excreted biomarkers may not directly reflect tissue-specific oxidative injury, and treatment-related changes in these markers do not establish the extent to which oxidative damage at the tissue or end-organ level is reversible [66]. Furthermore, oxidative-stress biomarkers are not currently used for routine longitudinal monitoring in pediatric OSA. Larger prospective studies using standardized sampling methods, validated pediatric reference ranges, and parallel assessment of tissue or end-organ outcomes are required before these biomarkers can be incorporated into clinical practice [67].

## 4. How Obstructive Sleep Apnea Induces Oxidative Stress

**Figure 2:**
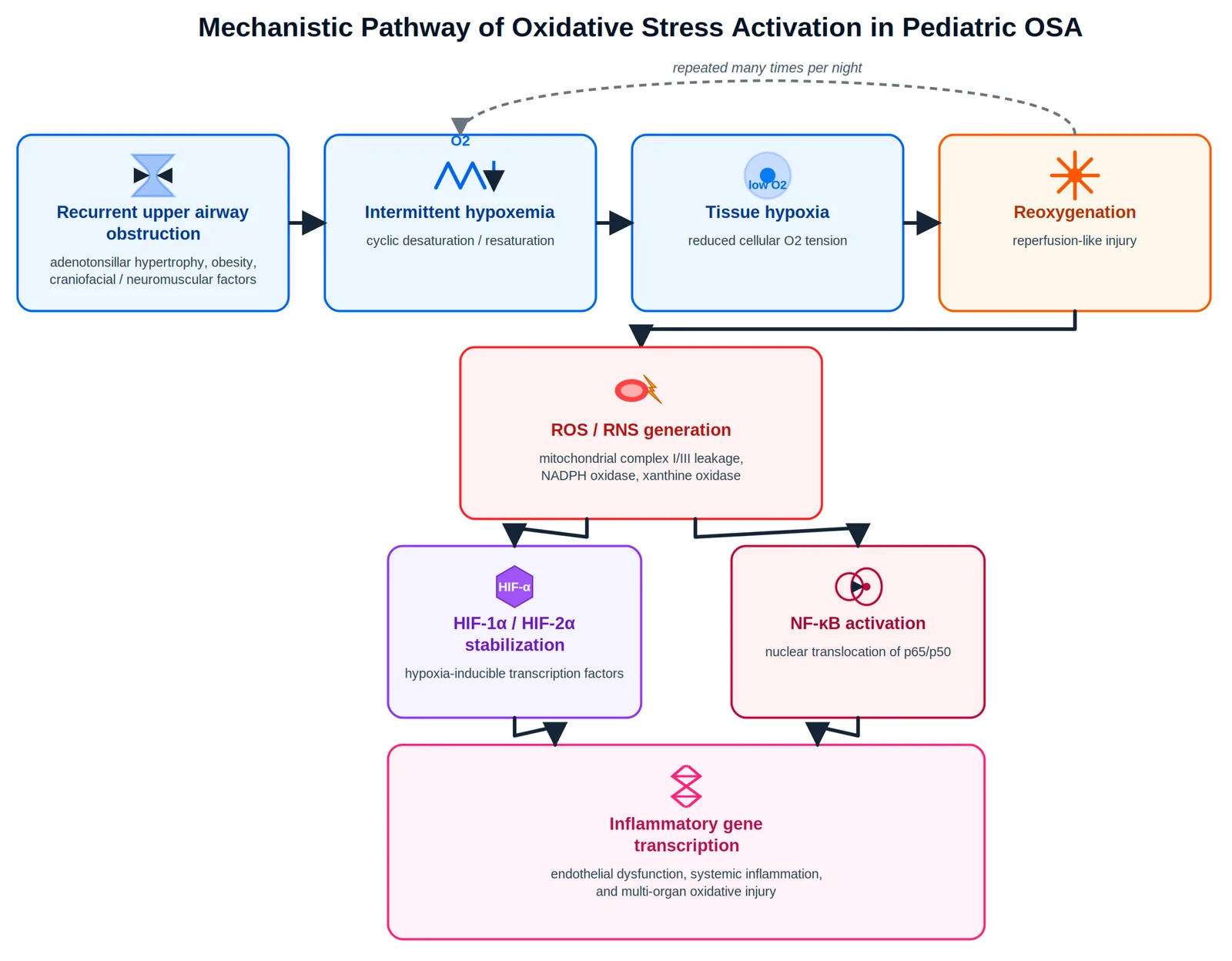
Mechanistic pathway linking recurrent upper airway obstruction, intermittent hypoxemia, tissue hypoxia, and reoxygenation to ROS/RNS generation, HIF-1α/HIF-2α signaling, NF-κB activation, and inflammatory gene transcription in pediatric OSA. Abbreviations: OSA, obstructive sleep apnea; ROS, reactive oxygen species; RNS, reactive nitrogen species; NADPH, nicotinamide adenine dinucleotide phosphate; HIF-1α, hypoxia-inducible factor 1-alpha; HIF-2α, hypoxia-inducible factor 2-alpha; NF-κB, nuclear factor kappa B; p65/p50, NF-κB transcription factor subunits. The figure was generated with the assistance of ChatGPT 5.6 Sol (OpenAI, Ohio, DE, USA) and subsequently reviewed and edited by the authors for scientific accuracy.

In pediatric OSA, recurrent upper airway obstruction first produces intermittent hypoxemia, resulting in repeated episodes of tissue and cellular hypoxia. Restoration of airflow is followed by reoxygenation, during which reactive oxygen species (ROS) generation increases sharply, producing a pattern comparable to ischemia–reperfusion injury. This reoxygenation-associated oxidative burst represents a key upstream event in the pathophysiological cascade. Increased ROS production, particularly from mitochondria and NADPH oxidases, subsequently activates redox-sensitive transcriptional pathways, including NF-κB and hypoxia-inducible factor signaling. Repeated hypoxia–reoxygenation favors HIF-1α-dependent pro-oxidant responses while altering the balance between HIF-1α and HIF-2α signaling, further amplifying oxidative stress. NF-κB activation then promotes the transcription of pro-inflammatory mediators, contributing to systemic inflammation, endothelial dysfunction, and subsequent end-organ injury. Once established, oxidative stress and inflammation can reinforce one another through secondary positive-feedback mechanisms, but both arise downstream of the initial hypoxia–reoxygenation insult [11,68]. This sequence is summarized in Figure 2.

These three HIF-α isoforms have distinct and partly opposing roles in the response to intermittent hypoxia. HIF-1α is broadly expressed and represents a major mediator of the acute hypoxic response. In OSA-related intermittent hypoxia, HIF-1α activity increases and promotes the expression of pro-oxidant pathways, including NADPH oxidases, while interacting with NF-κB-dependent signaling to amplify inflammatory responses. Increased HIF-1α activity has also been implicated in impaired glucose metabolism and insulin resistance [68]. In contrast, intermittent hypoxia may reduce HIF-2α activity, shifting the HIF-1α/HIF-2α balance toward a more pro-oxidant state. HIF-2α regulates antioxidant defenses as well as lipid and glucose metabolism, and its effects in adipose tissue appear to be context-dependent; experimental evidence suggests that it may partially counteract HIF-1α-mediated inflammation and metabolic dysfunction [69]. HIF-3α adds a further regulatory layer. Several HIF-3α splice variants, particularly inhibitory PAS domain protein (IPAS), can attenuate HIF-1α- and HIF-2α-dependent transcription through competition for HIF-β or formation of transcriptionally inactive complexes. Thus, differences in the relative activity of HIF-1α, HIF-2α, and inhibitory HIF-3α variants may contribute to the heterogeneous oxidative, inflammatory, and metabolic responses observed among patients exposed to similar degrees of intermittent hypoxia [70].

Because children with comparable apnea-hypopnea indices may show markedly different oxidative and end-organ injury, hypoxic-burden metrics—the oxygen-desaturation index, saturation nadir, and cumulative time below threshold—may better capture biological stress than event counts alone [71,72]. This distinction matters clinically: two children with identical apnea-hypopnea indices may experience very different patterns of desaturation depth and duration, and it is plausibly this hypoxic dose, rather than the number of discrete respiratory events, that determines the magnitude of redox injury. Chronic intermittent hypoxia can suppress nuclear factor erythroid 2-related factor 2 (Nrf2) signaling, which normally activates genes encoding antioxidant and detoxification enzymes, thereby reducing antioxidant-enzyme expression while simultaneously increasing ROS generation—a reciprocal failure of protection and production [73]. Because Nrf2 regulates several key antioxidant enzymes, its suppression during chronic intermittent hypoxia may contribute to the reduced antioxidant reserve observed in some pediatric cohorts [51,70]. Intermittent hypoxia, sympathetic activation, and sleep fragmentation should be considered interconnected rather than fully independent mechanisms of oxidative injury in OSA. Intermittent hypoxia appears to act as an important upstream stimulus: hypoxia–reoxygenation increases ROS generation and alters HIF-1α/HIF-2α signaling, while ROS-dependent enhancement of the carotid-body chemoreflex and attenuation of baroreflex activity promote sustained sympathetic outflow [68]. Experimental and clinical evidence further indicates that hypoxemia is an important determinant of acute sympathetic activation; in adults with OSA, prevention of desaturation by hyperoxia markedly attenuated apnea-associated sympathetic surges despite persistence of arousals. Pediatric studies similarly demonstrate increased catecholaminergic activity in children with sleep-disordered breathing, although the relative quantitative contribution of sympathetic activation versus direct hypoxia–reoxygenation to oxidative stress remains undefined [74].

Sleep fragmentation represents an additional and partly independent pathway. Experimental sleep-fragmentation models demonstrate increased oxidative stress and lipid peroxidation in the hippocampus, thalamus, and cerebral cortex, together with impaired antioxidant defenses [75]. In children with OSA, respiratory-related arousal frequency has also been independently associated with impaired endothelial function, supporting a contribution of sleep fragmentation beyond oxygen desaturation, although direct pediatric studies linking fragmentation specifically to oxidative biomarkers remain limited [76].

Mitochondrial injury accumulates in parallel: reduced mitochondrial DNA copy number has been reported in OSA and proposed as a marker of cumulative damage to mitochondrial proteins and impaired oxidative phosphorylation [77], though most mechanistic data linking mitochondrial dysfunction to impaired ventilatory control and upper-airway muscle function derive from adult, animal, or in vitro studies, with pediatric evidence still limited [78]. This gap is notable because impaired mitochondrial bioenergetics in upper-airway dilator muscles could, in principle, create a vicious cycle in which oxidative injury itself weakens the very muscles responsible for maintaining airway patency. Endoplasmic reticulum stress, triggered by the accumulation of misfolded proteins under oxidative conditions, has been mechanistically tied to impaired synaptic plasticity and memory; its pharmacological attenuation reverses these deficits in experimental models, supporting a causal rather than incidental role in the neurocognitive consequences of intermittent hypoxia [79].

Endothelial dysfunction represents a convergence point for these pathways and is frequently used as a functional readout of cumulative vascular oxidative injury, because it can be assessed non-invasively and responds dynamically to changes in disease severity or treatment; recovery after adenotonsillectomy or other effective treatment supports at least partial reversibility, though the magnitude varies with adherence, disease duration, obesity, and pre-existing vascular damage [80,81]. Endothelial function can deteriorate acutely and in a severity-dependent manner over a single night of obstructed breathing [82], and flow-mediated dilation is inversely related to apnea-hypopnea severity in children with OSA compared with controls [83]. Increased methylation of the endothelial nitric oxide synthase promoter, which reduces its expression, suggests that recurrent nocturnal stress produces longer-lasting epigenetic vascular alterations that may not fully normalize even after apparently successful treatment of the underlying respiratory disorder [84]. Collectively, these mechanisms converge on nitric oxide dysregulation, vascular smooth-muscle activation, and extracellular-matrix remodeling as shared downstream effectors of endothelial injury in OSA [85].

## 5. Biomarkers of Oxidative Stress in Pediatric OSA

Comparability across pediatric oxidative-biomarker studies is limited by differences in age, body mass index, pubertal stage, disease severity, biological sample type (serum, urine, exhaled breath), collection timing, and analytical technique—all of which restrict direct cross-study comparison [86,87,88]. This methodological heterogeneity means that apparently conflicting results across studies may partly reflect differences in study design rather than genuine biological inconsistency, complicating efforts to establish reference ranges or clinically actionable cut-offs [86,87,88]. Meta-analytic data show elevated lipid-peroxidation products, particularly malondialdehyde (MDA) and thiobarbituric acid-reactive substances, though most included studies involved adults [89]. MDA nonetheless remains among the most consistently OSA-associated markers across systematic evaluations [90], and circulating 8-isoprostane, a marker of non-enzymatic lipid peroxidation, has also been associated with severity and contributes to vascular dysfunction, again predominantly in adult cohorts [91].

Protein and DNA oxidation markers show a more heterogeneous pattern than lipid-peroxidation products, and their interpretation requires particular caution given their sensitivity to sample handling and storage [92]. In adults without major comorbidities, protein carbonyls did not differ significantly from controls, while glutathione redox balance was more consistently altered: overnight reductions in total antioxidant status and the GSH/GSSG ratio suggest consumption of antioxidant reserves during hypoxia-reoxygenation rather than a stable baseline deficit [92]. Advanced oxidation protein products are generally elevated in OSA but with substantial interstudy variability, again largely from adult data [89]. Urinary 8-OHdG correlates more strongly with oxygen-desaturation indices than with the apnea-hypopnea index, reinforcing the broader theme that hypoxic burden, rather than event count, best predicts oxidative injury [93]. As noted above, it also correlates with cognitive dysfunction, suggesting that oxidative DNA injury contributes specifically to neurocognitive morbidity rather than representing only a non-specific systemic signal [94,95]. Antioxidant-enzyme data are similarly inconsistent: meta-analyses show no uniform superoxide dismutase pattern across pediatric and adult studies combined [90], and catalase activity does not differ significantly in uncomplicated pediatric OSA despite altered glutathione status, indicating that antioxidant-enzyme responses are not uniformly suppressed but rather selectively and variably affected [92]. Importantly, antioxidant biomarkers are influenced by biological timing. GSH/GSSG redox status and measures of global antioxidant capacity show diurnal variation and may also be affected by meals, sleep–wake state, and recent oxidative exposures [95,96]. Consequently, differences between evening and morning values cannot necessarily be attributed entirely to nocturnal OSA-related oxidative stress. This temporal variability limits the diagnostic utility of isolated TAC or GSH/GSSG measurements and reinforces the need for standardized fasting status and sampling time. Paired measurements obtained under identical temporal conditions may be more informative for longitudinal assessment or treatment-response studies than single absolute values [96].

Inflammatory biomarkers closely track this oxidative signature: serum IL-6 and IL-8 are increased in children with OSA [97,98], and morning TNF-α correlates with respiratory-event severity and sleep fragmentation. Genetic TNF-related variants modulate the phenotype and may explain why some children develop more pronounced inflammatory responses than others despite similar respiratory severity [99,100]. CRP and high-sensitivity CRP are elevated, particularly in moderate-to-severe or residual disease, and decrease following treatment, supporting their potential use as adjunct markers of treatment response [101,102,103]. Serum from children with OSA activates NF-κB and nuclear p65 translocation in experimental systems, supporting both local and systemic pathway activation and providing mechanistic evidence that circulating factors, not merely direct tissue hypoxia, propagate inflammatory signaling [104]. Emerging candidates such as cathepsin Z and sex-hormone-binding globulin show promising diagnostic performance pending independent validation [105], and disulfide homeostasis shifts toward oxidation that improve after adenotonsillectomy suggest that treatment-related biomarker trajectories may prove more informative than isolated baseline values [106]. Metabolomic profiling may broaden biomarker assessment beyond individual oxidative molecules. Urine is particularly attractive in children because it can be obtained repeatedly and non-invasively. Untargeted urinary metabolomics in pediatric OSA has identified distinct metabolic profiles involving amino-acid, carbohydrate, microbial, and lipid pathways [107], although current studies remain small and require external validation. Exhaled-breath mass spectrometry represents another promising non-invasive approach: recent pediatric work has identified breath molecular features associated with urinary 8-iso-PGF2α [86], suggesting that breath profiling may eventually provide real-time information on systemic or airway oxidative stress. However, such techniques remain exploratory and are not currently suitable for routine clinical diagnosis [107].

Overall, the biomarker literature reviewed in this section suggests that oxidative and inflammatory pathways in pediatric OSA are closely interlinked rather than independent processes [70,104,106]. This has practical implications for future biomarker panel design, as combining a small number of complementary oxidative and inflammatory markers may ultimately provide greater diagnostic or prognostic value than any single biomarker used in isolation [104,106].

## 6. Organ Damage Associated with Oxidative Stress

### 6.1. Cardiovascular and Endothelial Dysfunction

Evidence of early myocardial structural and functional change and elevated right-heart pressure exists, though the strength of evidence and the amount of longitudinal pediatric data remain limited [108]. Inflammatory and oxidative markers such as IL-6 and IL-8 are associated with reduced carotid distensibility and increased arterial stiffness even before overt vascular remodeling is detectable, suggesting these changes may precede measurable wall thickening and could represent an early, potentially reversible stage of vascular injury [109]. Moderate-to-severe OSA independently raises the risk of nocturnal hypertension regardless of obesity [110], an association confirmed across meta-analyses despite methodological heterogeneity [111]. Both oxygen-desaturation burden and adiposity independently predict hypertension [112], with pro-oxidant and inflammatory mechanisms converging on nitric oxide dysregulation, vascular smooth-muscle activation, and accelerated atherosclerotic pathways [113]. Taken together, these findings suggest that cardiovascular risk in pediatric OSA begins as a functional, biochemically detectable process well before it becomes structurally apparent on conventional imaging [108,109,110,111,112,113].

### 6.2. Neurocognitive and Behavioral Consequences

Oxidative stress is directly implicated in the neurocognitive dysfunction associated with OSA, plausibly through oxidative injury to brain regions involved in memory and behavioral regulation—particularly the hippocampus and prefrontal cortex, which appear especially vulnerable to intermittent hypoxia given their high metabolic demand [114]. Memory impairment, assessed with standardized verbal learning tests, correlates with indices of nocturnal hypoxia, supporting a link between oxygen instability and cognitive dysfunction [115,116]. Age-dependent associations exist between OSA features and coexisting ADHD, illustrating that behavioral manifestations vary with developmental stage [117]. Expressive vocabulary and behavioral problems are more common in preschool OSA and improve variably after adenotonsillectomy [118], while executive dysfunction—affecting inhibition, working memory, and cognitive shifting—does not correlate linearly with disease severity [119]. This reinforces the broader pattern seen throughout this review, in which oxidative and inflammatory burden, rather than the apnea-hypopnea index alone, appears to better track functional impairment [86,115,116,117,118,119].

### 6.3. Metabolic Dysfunction and Insulin Resistance

OSA is associated with higher fasting insulin and HOMA-IR, strongly modified by sleep duration, age, pubertal stage, and adiposity, indicating that metabolic risk in pediatric OSA cannot be interpreted independently of weight status [120]. Treatment improves metabolic and inflammatory markers to a degree that depends on obesity status [121], and can improve lipid parameters independently of weight loss, suggesting a direct metabolic benefit of resolving intermittent hypoxia rather than a purely weight-mediated effect [122]. These findings collectively argue against treating pediatric OSA-related metabolic risk as simply a downstream consequence of obesity, since hypoxia-specific effects appear to operate at least partly independently of body weight [120,121,122]. In obese adolescents, OSA is associated with elevated transaminases and more advanced hepatic fibrosis [123], consistent with a “multiple-hit” model in which obesity and insulin resistance establish steatosis while intermittent hypoxia and oxidative stress accelerate inflammation and fibrosis [124]. Aminotransferase elevation concentrates in overweight or obese children with OSA [125], and positive airway pressure therapy improves ALT, metabolic markers, and urinary F2-isoprostanes despite unchanged or increased body mass index, again pointing to a hypoxia-specific rather than purely weight-dependent mechanism [126].

### 6.4. Renal Involvement and Subclinical Kidney Injury

Renal involvement has also been investigated in OSA, although the strength of evidence differs substantially between pediatric and adult populations. In children, moderate-to-severe OSA has been associated with increased low-grade albuminuria, with urinary albumin excretion correlating with nocturnal oxygen desaturation and respiratory arousals [127]. In contrast, evidence for elevations in cystatin C, NGAL, and IL-18 derives predominantly from adult studies and meta-analyses, where these markers have been associated with OSA severity and hypoxic burden. Therefore, although these biomarkers suggest a potential pathway of subclinical glomerular and tubular injury, their clinical relevance in pediatric OSA remains insufficiently established [128].

### 6.5. Immune and Inflammatory Dysregulation

Immunologically, children with OSA show an altered CD4+/CD8+ ratio alongside increased IL-4, IL-6, IL-10, interferon-γ, IgA, and complement C3, indicating combined cellular and humoral change [129]. These cytokines correlate with attention and executive-function measures, suggesting a link between immune activation and neurocognitive morbidity that may share common upstream oxidative triggers [130]. Taken together with the cardiovascular, metabolic, and hepatic findings above, this pattern of renal and immune involvement completes a picture of pediatric OSA as a genuinely multisystem disease, in which oxidative stress and low-grade inflammation act as shared upstream drivers across seemingly unrelated organ systems, rather than each organ being affected through an independent mechanism [114,128,129,130] (Table 1).

## 7. Conclusions

Pediatric obstructive sleep apnea is increasingly recognized as a systemic disorder in which oxidative stress functions as a central pathophysiological hub connecting intermittent hypoxia and sleep fragmentation to cardiovascular, neurocognitive, metabolic, hepatic, renal, and immune morbidity. Repeated hypoxia-reoxygenation cycles overwhelm antioxidant defenses and activate redox-sensitive signaling—HIF-1α, NF-κB, NADPH oxidases, endoplasmic reticulum stress—establishing self-perpetuating feedback loops of injury and inflammation that extend well beyond the upper airway itself.

Organ damage does not track the apnea-hypopnea index alone: children with similar polysomnographic severity can show markedly different oxidative injury, reflecting individual differences in hypoxic burden, obesity, genetic background, antioxidant capacity, and disease duration [50,68,69]. This mismatch between a single respiratory metric and the true biological burden of disease is arguably the central clinical problem this review has sought to characterize, and it recurs across nearly every organ system discussed above—from endothelial function to hepatic and renal injury markers. Although numerous oxidative and inflammatory biomarkers show promising associations with pediatric OSA, none yet possesses sufficient diagnostic accuracy or standardization for routine clinical use, owing to methodological heterogeneity and the confounding influence of obesity [86,87,88].

Because most affected children have not yet developed irreversible cardiovascular or metabolic disease, many oxidative and inflammatory alterations may remain reversible with prompt recognition and treatment [16,80,81]—underscoring the value of early diagnosis and individualized, longitudinal follow-up, particularly in children with severe hypoxemia, obesity, or residual OSA after adenotonsillectomy. Clinicians should be aware that a reassuring apnea-hypopnea index does not necessarily indicate low biological risk, and that persistent symptoms, obesity, or known genetic predisposition may warrant closer surveillance even when polysomnographic severity appears modest [50].

Translating this evidence into practice will require several parallel advances. Because the discordance between apnea-hypopnea-index-based severity and measurable oxidative or end-organ injury is a recurring theme throughout this review, hypoxic-burden metrics and inflammatory or oxidative biomarkers could complement conventional polysomnographic scoring in identifying children who warrant closer follow-up, even when the apnea-hypopnea index alone appears reassuring [50,68,69]. Realizing this potential is nonetheless constrained by the fact that most available pediatric biomarker studies are cross-sectional, single-center, and rely on heterogeneous assay platforms, which limits their comparability and delays the establishment of pediatric-specific reference ranges [86,87,88,89,90]. Longitudinal cohorts that track oxidative and inflammatory trajectories before and after treatment, ideally alongside hypoxic-burden metrics rather than the apnea-hypopnea index alone, would help clarify which biomarkers genuinely predict long-term organ risk rather than merely reflecting acute hypoxic exposure.

Emerging proteomic and metabolomic approaches, including candidate markers such as cathepsin Z and sex-hormone-binding globulin, illustrate the potential of untargeted discovery platforms to identify pediatric-specific signatures that may outperform single conventional markers, although independent validation in larger, more diverse cohorts remains necessary before any of these candidates can be considered for clinical use [106]. Combining a small panel of complementary oxidative and inflammatory markers, rather than relying on any single analyte, may ultimately provide better discrimination of disease severity and treatment response, consistent with the interlinked nature of oxidative and inflammatory pathways described throughout this review [105,107]. Because obesity is both a major confounder and an increasingly common coexisting condition in pediatric OSA, future studies should stratify analyses by adiposity and pubertal stage, or specifically study non-obese children, to isolate the oxidative and inflammatory contribution of intermittent hypoxia itself from that of excess adiposity [24,25,26]. Interventional studies that pair adenotonsillectomy or positive airway pressure therapy with pre- and post-treatment biomarker sampling could further clarify which oxidative alterations are reversible and which may persist, informing decisions about the intensity and duration of postoperative surveillance in children with residual risk factors [16,80,81,126].

An additional emerging area concerns the interaction between pediatric OSA, mouth breathing, oral health, and the upper-airway microbiome. Mouth breathing is common in pediatric OSA and may alter the local oral environment, while pediatric studies have reported differences in oral and nasal microbial composition between children with OSA and controls [131,132]. Children with OSA may also exhibit greater plaque accumulation, gingival inflammation, altered oral pH, and higher cariogenic bacterial counts, although the strength and consistency of these associations remain variable [133]. These findings raise the possibility that local microbial dysbiosis and oral inflammatory processes may interact with the oxidative and inflammatory pathways already implicated in OSA. Saliva is particularly attractive as a non-invasive sampling matrix, and future studies integrating salivary inflammatory and oxidative biomarkers with metabolomic, proteomic, and microbiome profiling (“salivaomics”) may help identify pediatric-specific signatures of disease burden and treatment response [134].

From a practical standpoint, clinicians managing children with OSA should not rely on the apnea-hypopnea index in isolation when counselling families about long-term risk, particularly in the presence of obesity, prolonged or severe desaturation, or a known genetic predisposition to impaired antioxidant capacity; in such children, a lower threshold for closer cardiovascular, metabolic, and neurocognitive surveillance appears justified even when polysomnographic severity is classified as mild [4,6,7,8,9,10,50]. At the research level, priority should be given to multicenter pediatric cohorts using harmonized, pre-specified biomarker panels and standardized sample-handling protocols, since much of the heterogeneity described throughout this review likely reflects methodological rather than genuine biological variability [86,87,88]. Incorporating genomic and epigenetic data, such as variation in antioxidant-enzyme genes or eNOS promoter methylation, alongside oxidative and inflammatory biomarkers may eventually allow risk stratification that accounts for individual susceptibility rather than respiratory-event count alone [15]. Ultimately, demonstrating that biomarker-guided intervention improves long-term cardiovascular, metabolic, or neurocognitive outcomes—not merely that biomarkers correlate with disease severity—will be the decisive step in moving this field from mechanistic description toward clinical application.

## Figures and Tables

**Table 1:** Oxidative stress and inflammation-related biomarkers in pediatric obstructive sleep apnea and their impact on organ function. ↑ indicates increased levels/activity; ↓ indicates decreased levels/activity.

Biomarker/Pathway	Change in Pediatric OSA	What It Reflects	Main Organ/Function Affected	Reported Functional Impact/Clinical Significance	Evidence Type & Reference
Malondialde-hyde (MDA)	↑	Lipid peroxidation and oxidative membrane injury	Systemic/cardiovascular	Indicates increased oxidative burden; among the most consistently OSA-associated oxidative markers, although much of the evidence derives from adult studies.	Mixed (re-views/meta-analyses) [87,88,89]
Thiobarbituric acid-reactive substances (TBARS)	↑	Lipid peroxidation	Systemic/vascular	Supports increased oxidative damage associated with repetitive hypoxia-reoxygenation.	Mixed (re-views/meta-analyses) [87,88,89]
8-Isoprostane/F2-isoprostanes	↑	Non-enzymatic lipid peroxidation	Cardiovascular/endothelial; metabolic-hepatic	Associated with OSA severity and vascular dysfunction; urinary F2-isoprostanes may improve following positive airway pressure therapy.	Pediatric [57,86,126]
Protein carbonyls	No consistent increase	Oxidative modification of proteins	Systemic	Pediatric studies without major comorbidities did not demonstrate significant differences from controls, illustrating biomarker heterogeneity.	Mixed (adult-predominant) [87,88,91]
Advanced oxidation protein products (AOPP)	Generally ↑, but variable	Protein oxidation	Systemic/vascular	Suggest oxidative protein injury, although substantial interstudy variability limits interpretation.	Mixed (adult-predominant) [87,88,90]
GSH/GSSG ratio	↓ overnight	Impaired glutathione redox balance and consumption of antioxidant reserves	Systemic/cellular	Suggests depletion of antioxidant capacity during repeated hypoxia-reoxygenation rather than a fixed baseline antioxidant deficiency.	Adult [91,95,96]
Total antioxidant status	↓ overnight	Global antioxidant defense capacity	Systemic	Indicates consumption of endogenous antioxidant reserves during nocturnal obstructive events.	Adult [92,96]
Superoxide dismutase (SOD)	Inconsistent	Enzymatic antioxidant defense	Systemic/vascular	No uniform pattern across studies; therefore, currently unsuitable as a stand-alone clinical biomarker.	Mixed (adult-predominant) [60,87,88,89]
Catalase	Often unchanged	Hydrogen-peroxide detoxification	Systemic	May remain preserved despite altered glutathione homeostasis, indicating selective rather than uniform impairment of antioxidant defenses.	Mixed (adult-predominant) [87,88,89]
Urinary 8-hydroxy-2′-deoxyguanosine (8-OHdG)	↑	Oxidative DNA damage	Neurocognitive	Correlates more strongly with oxygen-desaturation indices than with AHI and is associated with cognitive dysfunction, supporting a relationship between oxidative DNA injury and neurocognitive morbidity.	Pediatric [63]
IL-6	↑	Systemic inflammation closely linked to oxidative stress	Cardiovascular/vascular; immune; neurocognitive	Associated with reduced carotid distensibility and increased arterial stiffness; immune activation may also relate to attention and executive-function abnormalities.	Pediatric [26,97,98]
IL-8	↑	Systemic inflammatory response	Cardiovascular/vascular	Associated with reduced carotid distensibility and increased arterial stiffness, suggesting early vascular injury before overt structural remodeling.	Pediatric [97,98]
TNF-α	↑	Redox-related systemic inflammation	Neurobehavioral/systemic inflammatory response	Morning levels correlate with respiratory-event severity and sleep fragmentation; genetic variants may modulate individual inflammatory susceptibility.	Pediatric [99,100]
CRP/high-sensitivity CRP	↑	Systemic low-grade inflammation	Cardiovascular/systemic	Particularly elevated in moderate-to-severe or residual OSA and may decline following treatment, supporting potential use for treatment monitoring.	Mixed (mostly pediatric) [6,26,101,102,103]
NF-κB activa-tion/nuclear p65 transloca-tion	↑	Redox-sensitive pro-inflammatory signaling	Multisystem	Demonstrates systemic inflammatory pathway activation and provides a mechanistic bridge between circulating factors, oxidative stress, and organ injury.	Pediatric [104]
Thiol-disulfide homeostasis	Shift toward oxidation	Systemic redox imbalance	Systemic/vascular	Oxidative shift may improve after adenotonsillectomy, suggesting usefulness for monitoring treatment-related changes.	Pediatric [106]
Cathepsin Z	Emerging biomarker	Proteomic signature associated with pediatric OSA	Systemic	Shows potential diagnostic performance but requires independent validation before clinical use.	Pediatric [105]
Sex-hormone-binding globulin (SHBG)	Emerging biomarker	Metabolic/proteo-mic alteration	Metabolic	Potential diagnostic biomarker, but clinical significance remains to be validated.	Pediatric [105]
Fasting insulin/HOMA-IR	↑	Insulin resistance associated with OSA-related metabolic stress	Metabolic	Reflect metabolic dysfunction; associations are strongly influenced by adiposity, age, pubertal stage, and sleep duration.	Pediatric [69,120]
ALT/transam-inases	↑ particularly with obesity	Hepatic injury in the setting of intermittent hypoxia and metabolic dysfunction	Hepatic	Associated with more advanced hepatic inju-ry/fibrosis in obese adolescents; improvement after PAP despite unchanged BMI supports a hypoxia-specific component.	Pediatric [123,124,125,126]
Cystatin C	↑	Renal functional impairment	Renal	Supports early renal involvement within the cardio-vascular-renal continuum associated with hypoxic severity and duration.	Mixed (pediatric + adult) [127]
NGAL	↑	Tubular renal injury	Renal	Indicates subclinical kidney injury, particularly in more severe disease.	Mixed (pediatric + adult) [127]
Urinary IL-18	↑	Renal inflammatory/tubular injury	Renal	Suggests inflammatory renal involvement associated with OSA severity and hypertension.	Pediatric/mixed [127]
CD4+/CD8+ ratio and cytokine profile (IL-4, IL-6, IL-10, IFN-γ)	Altered/↑ cytokines	Cellular and humoral immune activation	Immune/neurocognitive	Cytokine alterations correlate with attention and executive-function measures, suggesting interaction between immune activation, oxidative stress, and neurocognitive morbidity.	Pediatric [121,128,129]

## Data Availability

No new data were created or analyzed in this study. Data sharing is not applicable to this article.
